# Reduction of Dislocations of the Hip-Joint by Manipulation

**Published:** 1858-02

**Authors:** Frank Hastings Hamilton


### BUFFALO MEDICAL JOURNAL

AND

### MONTHLY REVIEW.

VOL. 13. FEBRUARY, 1858. NO. 9.

### ORIGINAL COMMUNICATIONS.

ART. I.—*Reduction of Dislocations of the Hip-Joint by Manipulation. Fragments of the Literature of this method.* Compiled by FRANK HASTINGS HAMILTON, M. D.

(Continued from Page 330.)

"A primary luxation directly backwards was brought into the Pennsylvania Hospital last winter, and after various attempts to reduce it by powerful extension, in the different directions before described, Dr. Physick thought it probable that the head of the bone had slipped through a slit, or longitudinal rent, in the capsular ligament, and that this rent embraced it at the neck, as a button hole does a button; under this impression, after the extension had ceased, he made an abduction of the thigh, pushing at the same time the head of the bone forwards towards the acetabulum; this manœuvre in a moment succeeded without any more force than the Dr. was himself able to exert—a strong confirmation of his opinion respecting the nature of the obstacle previously existing. In all difficult cases it will be proper to try every possible motion of the limb, before abandoning the case as hopeless; very often, after great force has failed, a gentle effort in some new direction is found successful."—*Elements of Surgery. By John Syng Dorsey, M.D. Philadelphia*, 1813, *vol.* i, *p.* 242.

This is, no doubt, the same case which has been described by Dr. James L. Phelps, of New York, who was himself a witness of the reduction. His account of the mode of procedure is taken from his notes written at the time, and is quite minute.

The patient, an athletic man, 40 years of age, had luxated his femur upwards and backwards, five hours before, on the 7th of Jan., 1811. Dr. Physick made four successive attempts to reduce it by the ordinary modes of extension and counter-extension, during which time the patient was bled from both arms to fainting. These procedures had occupied more than an hour, and the united strength of eight men had been employed. After the last effort, Dr. Physick having waited a moment contemplating his exhausted patient, "took hold of the limb and brought the thigh up to a little more than a right angle with the body, and then gave it an outward circular sweep." At this moment the bone slipped into its socket with a visible muscular action.—*Transactions of the New York State Medical Society, for* 1856, *p.* 169 *et seq.*

Before describing the method recommended and practised by Dr. Nathan Smith, it will be proper to give a brief epitome of the history of this distinguished physician and surgeon; especially since in the writings of his no less distinguished son, Dr. Nathan R. Smith, of Baltimore, will be found the most minute and elaborate description of the method of reducing dislocations of the hip by manipulation, which up to that day, had been written, and the discovery of which method is by the son attributed to his father, Dr. Nathan Smith.

He was born in Rehoboth, Mass., on the 30th of Sept., 1762. While he was yet young, the family removed to Chester, Windsor County, Vermont, where his parents remained until their death. His early life was passed upon a farm, and chiefly in agricultural pursuits; but before he arrived at the period of manhood, he joined a body of Vermont militia, enlisted to repel the incursions of the bordering tribes of Indians. At the age of 27 years, having already acquired a thorough academical education, he commenced the study of medicine in the office of Dr. Goodhue, of Putney, Vt. He subsequently received the degree of Bachelor of Medicine from the University of Harvard, at Cambridge.

In —— Dr. Smith founded a medical department in connection with

Dartmouth College, at Hanover, in New Hampshire. Soon after he visited Great Britain, spending about a year in attendance upon the lectures at Edinburgh, and in the hospitals of London. On his return he continued to lecture at Dartmouth until the year 1813, when he accepted a professorship in the Medical Institution of Yale College, at New Haven, Ct., where he taught until the period of his death, which occurred in 1828. He was 66 years old when he died.*

"It is believed," says his son, "that he was the first in this country to perform the bold operation of extirpating the ovarian tumor. He was also the first to perform, in this country, the operation of staphyloraphy. He invented a new apparatus for the treatment of fractures. His mode of reducing dislocations of the hip is new, philosophical, and ingenious."

The only written account of his views in relation to the reduction of dislocations of the hip, which I can find given by himself, is contained in a pamphlet entitled "*Report of the trial of an Action. Charles Lowell against John Faxon and Macajah Hawks, Doctors of Medicine, defendants, for malpractice in the capacity of Physicians and Surgeons, at the Supreme Court of Maine, holden at Machias, for the County of Washington, June term,* 1824. *Before the Hon. Nathan Weston, Jun., Justice of the Court.*"

This was the third trial of the same action, and the case being one of great importance to all parties, the depositions of several distinguished surgeons were obtained, among which we find the deposition of Dr. Nathan Smith, and so much of which as is pertinent to the object of this paper, I shall quote:

"I do not think that the mechanical powers, such as the wheel and axle, or the pulleys are necessary to reduce a dislocated hip, or any other dislocation. They have sometimes been used with effect, but they have oftener been injurious, and what can be effected with them can be effected without them. It is not the quantum of force which reduces the dislocated bones, so much as it is the direction of the force; and this can be given by the hand of skill, better than by pulleys, &c. In reducing the hip-joint it cannot be done by direct pulling; but we take advantage of the thigh bone as a lever to move the head of the bone from the place where it may be lodged, and bring it into its former situation. In some cases the fulcrum is some of the bones of the pelvis; in others we have to supply it by some external body.

---

* Biographical Memoir. By Jonathan Knight, of New Haven.

"*Question by defendents' attorney.* Did you ever reduce a dislocated hip? and if so, please to state the manner.

"*Answer.* I once reduced a dislocated hip-joint. It was dislocated upward and backward; and after pulling it in every direction but the right, it was reduced easily by carrying the knee towards the patient's face. I had the assistance of two men only."

This deposition was made about four years before his death, and we cannot now ascertain whether he ever had another opportunity of reducing a dislocated hip, and of determining again the value of this method; nor do we know at what period of his life the case occurred to which he here alludes.

Dr. Samuel Shumway, of Essex Co., N. Y., in a communication made to the New York Medical Society at its annual meeting in Feb., 1854, reports a case in which he had reduced an upward and backward dislocation of the hip, by flexing the leg on the thigh, the thigh on the pelvis, and then carrying the limb diagonally to the opposite side, from whence it was brought outwards and downwards. Which method, he affirms, was taught him by Dr. Nathan Smith, while he was a student of medicine at Yale College, during the session of 1815-16. He had seen Dr. Smith demonstrate it to the class on the skeleton. — *Transac. of the N. Y. State Med. Soc. for* 1854, *p.* 55 *et seq.*

Many other physicians and surgeons in different parts of the country have described also to the writer a mode of reduction essentially the same as that which was recommended by Dr. Smith, and which, they affirm, was learned from him.

In the year 1831, Dr. Nathan R. Smith, of Baltimore, the son of Dr. Nathan Smith, and the present professor of surgery in the University of Maryland, published in an octavo volume some of the manuscript writings of his father, and also an account of what he believed to be his father's mode of reducing dislocations of the hip, for which latter he was, however, obliged to trust entirely to his memory. The essay is comprised in twenty pages, and is composed chiefly of arguments intended to prove that the muscles are the chief agents in producing these dislocations, and that they may also be employed efficiently in accomplishing their reduction. The action of the muscles is still further explained and illustrated by several wood cuts. From this essay I shall only quote the concluding pages.

"*Proposed Method of effecting the Reduction of the Os Femoris, Dislocated upon the Dorsum Ilii.*

"Professor Nathan Smith used to relate, in his surgical lectures, a case of dislocation of the os femoris on the dorsum ilii, in which he promptly succeeded by the mere force of hands, in effecting the reduction. Notes of this case unfortunately I am not able to discover among his papers. The principal facts, however, are fresh in my memory, and will undoubtedly be borne in mind by many who have listened to his instructions.

"After attempting the ordinary methods by extension, in vain, he bent the leg upon the knee, seized the leg, and using it as a lever, rotated the thigh a little outward. Then he gently abducted the thigh, and lastly flexed it freely on the pelvis, by carrying the knee toward the face of the patient. These movements instantly succeeded, and with but little effort of strength.

"A medical gentleman of Massachusetts, who had been a pupil of my father, saw a similar case of dislocation—practiced the same method, and succeeded with equal facility. A letter from him to professor Smith, detailing the particulars of the case, I once saw, but unfortunately it cannot be found.

"The case quoted above, in which professor Physick succeeded in effecting reduction by a gentle abduction of the thigh, was one of dislocation backwards, behind the posterior border of the acetabulum. The case in which my father succeeded, was one of dislocation on the dorsum ilii, and he accomplished the reduction by a compound movement. The free flexion of the thigh forward on the pelvis, would have the effect, by calling into exercise the gluteus maximus, to throw the head of the femur downwards, behind the border of the acetabulum, precisely in the position in which it was fixed in professor Physick's case. The rotation of the thigh outward, and the abduction of the member must, as can be anatomically demonstrated, have thrown the head of the bone forward into the acetabulum. When the bone is dislocated on the dorsum, the trochanter is dragged forward, the femur rotated inwards, and the head and neck of the bone are applied flatways on the pelvis. The rotation of the limb outward would, therefore, throw the trochanter outward, and cause the head to present in an attitude favorable to its entrance into the acetabulum. The abduction of the bone at the same moment, would have the effect to call powerfully into exercise the adductor muscles which come from the anterior part of the pelvis, and are inserted into the linea aspera. The strong effort of these muscles will necessarily tend, when this movement is made, to drag the head of the bone directly forward and downward, into the acetabulum. If we consider these muscles to represent the power, the hand of the surgeon (abduction being completed) applied to the knee, will be the fulcrum, or centre of motion, and the resistance is at the head of the bone. The adductor muscles will then act on a lever of the third kind, but, acting near its extremity and nearly at right angles to it, will exercise a power far greater than muscles ordinarily do—far greater than those muscles themselves do in the ordinary exercise of their functions. The effect of their contraction, then, as they will be much excited, must be very great, in aiding the reduction.

"If, while abduction is being made, we regard the hand of the surgeon at the knee as the power, then do the tense abductor muscles furnish the fulcrum, and the head of the bone, the resistance. The power exercised by the surgeon then operates upon far the longest arm of a lever of the first kind, and therefore with great mechanical advantage, in throwing the head of the bone toward the acetabulum.

"It should be particularly noticed that, while this compound movement of the bone thus calls into exercise, with their utmost power, the muscles which aid the reduction, it most effectually relaxes those (the glutei) which tend to resist it, and which were instrumental in effecting the dislocation. It will be observed that these, as well as all the other muscles inserted into the upper portion of the bone, must powerfully resist when the reduction is attempted by direct extension. Two very important objects then are at once accomplished: 1st, we call the adjuvant muscles most powerfully into exercise; 2d, we effectually avoid the resistance of those muscles, the action of which is by all surgeons regarded as the chief impediment to the reduction of all difficult dislocations.

"As I before remarked, the cases in which lateral movements with gentle force have succeeded, either by design or fortuitously, in promptly reducing dislocations which have resisted more powerful means, induce me to believe that there is a secret method in which we may uniformly thus succeed; and that method I believe to be the one in which the movements described above are employed. This I infer from facts which I have stated, and from the anatomical mechanism of the joint and appendages.

"The practical precepts derived from the above, are concisely these. The patient being prepared for the operation by whatever means may be deemed necessary, may be placed in an attitude convenient for the operation with the body securely fixed, by placing him in the horizontal posture, on a narnow table covered with blankets, and on the sound side. To the table his body should be firmly fixed, and this can be conveniently done by folding a sheet several times, length-ways—then applying the middle of the broad band thus made to the inner and upper part of the sound thigh—carrying its extremities under the table, crossing them beneath it, and then carrying them obliquely up and crossing them firmly over the trunk, above the injured hip. The ends may then be secured beneath the table. To support the trunk the more firmly, a pillow may be placed on each side of it upon the table, and be included in the bandage. Should the operator design to employ any degree of extension, a counter-extending band may be placed in the perinæum, and carried up to the extremity of the table, be fixed to some more firm body, or held by the hands of assistants.

"The operator now standing on the side to which the patient's back presents, grasps the knee of the dislocated member with his right hand, (if the left femur be dislocated—vice versa, if the right) and the ankle with the left. The first effort which he makes is to flex the leg upon the thigh, in order to make the leg a lever with which he may operate on the thigh-bone. The next movement is a gentle rotatian of the thigh outward, by inclining the foot toward the ground, and rotating the knee outward. Next the thigh is to be *slightly* abducted by pressing the knee directly outward. Lastly, the surgeon freely flexes the thigh upon the pelvis by thrusting the knee upward toward the face of the patient, *and at the same moment the abduction is to be increased.*

"Professor N. Smith regarded the free flexion of the thigh upon the pelvis as a very important part of the compound movement. He believed that it threw the head of the bone downward, behind the acetabulum, where the margin of the cup is less prominent, and over which, therefore, the adductor muscles would drag it with less difficulty into its place.

"The operator may slightly vary these movements, as he increases them, so as to give some degree of rocking motion to the head of the os femoris, which will thereby be disengaged with the more facility from its confined situation among the muscles.

"Should the operator deem it necessary to conjoin extension with these efforts, a band may be attached above the knee, in the usual manner. With this, extension is made horizontally—in the direction in which the bone presents. Then it is directed a little outward, so as to favor the necessary rotation and abduction. Lastly, it is made in a direction obliquely upward from the table, in order to favor the flexion upon the thigh. This last extending effort may be made by an assistant standing on the table, near its foot, with a band thrown round his neck and shoulder, and attached to the limb in the usual way. When the head of the bone is supposed to be approaching the cup, an assistant may press with his hands firmly upon the trochanter, downward and forward, toward the acetabulum, thus aiding the effort of the muscles.

"When the femur is dislocated backward into the ischiatic notch, abduction seems to be the only movement which is necessary, since the head of the bone is already behind the acetabulum. This was precisely the movement with which professor Physick succeeded in such a case.

"Of the other less difficult varieties of dislocation of the femur, it has not been my present purpose to treat."

(To be Continued.)

